# RNA polymerase III transcription as a disease factor

**DOI:** 10.1101/gad.333989.119

**Published:** 2020-07-01

**Authors:** Meghdad Yeganeh, Nouria Hernandez

**Affiliations:** Center for Integrative Genomics, Faculty of Biology and Medicine, University of Lausanne, 1015 Lausanne, Switzerland

**Keywords:** RNA polymerase III, transcription, disease, cancer, leukodystrophy, tRNA, RMRP, BC200, nc886, Alu

## Abstract

RNA polymerase (Pol) III is responsible for transcription of different noncoding genes in eukaryotic cells, whose RNA products have well-defined functions in translation and other biological processes for some, and functions that remain to be defined for others. For all of them, however, new functions are being described. For example, Pol III products have been reported to regulate certain proteins such as protein kinase R (PKR) by direct association, to constitute the source of very short RNAs with regulatory roles in gene expression, or to control microRNA levels by sequestration. Consistent with these many functions, deregulation of Pol III transcribed genes is associated with a large variety of human disorders. Here we review different human diseases that have been linked to defects in the Pol III transcription apparatus or to Pol III products imbalance and discuss the possible underlying mechanisms.

## Abstract (precis)

In this review, Yeganeh et al. summarize different human diseases that have been linked to defects in the Pol III transcription apparatus or to Pol III products imbalance and discuss the possible underlying mechanisms.

Among the three eukaryotic nuclear RNA polymerases (Pols), Pol I and Pol III transcribe noncoding RNAs involved in fundamental cellular processes. Pol I is responsible for the synthesis of all RNA components of the ribosome except for 5S RNA, which is synthesized by Pol III. Pol III transcribes in addition a collection of noncoding genes whose products are also involved in translation and translation control (tRNAs [transfer RNAs] and BC200), are part of the vault particle (vault RNAs), or are involved in various processes such as protein translocation as part of the signal recognition particle (7SL), regulation of Pol II elongation (7SK), or processing of other RNA molecules (U6, RPPH1 [ribonuclease P RNA component H1], and RMRP [RNA component of mitochondrial RNA processing endoribonuclease]) (Dieci et al. 2013). In addition, many genomic loci harbor Pol III promoter structures and are indeed transcribed either in a tissue-specific manner (BC200) or in certain malignancies (Alu RNAs and other loci). The function of many of these RNAs is not or poorly understood, and new functions are being discovered even for well-characterized Pol III products. Not unexpectedly, deregulation of Pol III transcription has been found to cause or to be linked to various diseases. Such deregulation can arise either from alterations within the basal Pol III transcription machinery, which can affect the synthesis of many Pol III products and thus have very pleiotropic effects, or from mutation of specific Pol III transcribed genes.

The basal Pol III transcription machinery comprises the enzyme itself and transcription factors that recruit Pol III to its different promoters. Most are composed of several subunits, many of which have been found mutated in certain diseases. Pol III is composed of 17 subunits (Fig. 1), of which five are shared among Pol III, Pol I, and Pol II, and two shared between Pol III and Pol I. Ten subunits are unique to Pol III (Vannini and Cramer 2012). Pol III recruitment to its target genes is mediated by several transcription factors, which vary according to the three main types of Pol III promoter structures. Type 1 and 2 promoters are gene-internal, whereas type 3 promoters are located in the 5′ flanking region. In type 1 promoters, Pol III is recruited to a DNA-bound complex composed of TFIIIA (transcription factor for RNA Pol III A), TFIIIC, and BRF1-TFIIIB (consisting of BDP1, BRF1, and TBP [TATA-binding protein]), while in type 2 promoters, TFIIIA is not required. In type 3 promoters, Pol III recruitment is mediated by SNAPc (snRNA-activating protein complex) and BRF2–TFIIIB (identical to BRF1-TFIIIB but for the replacement of BRF1 by BRF2) (Fig. 2; Schramm and Hernandez 2002).

**Figure 1.**
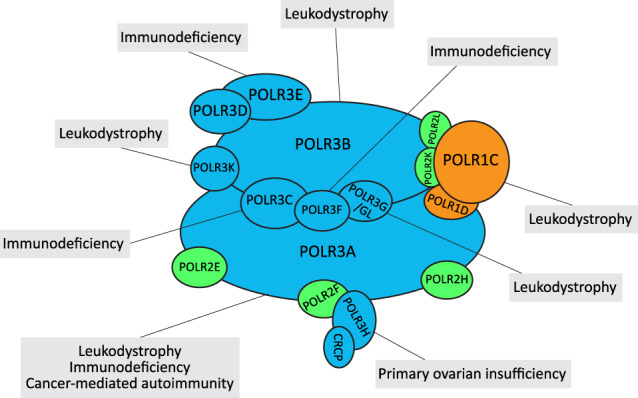
Pol III subunits: Subunits shared among Pol III, Pol I, and Pol II are shown in green; subunits shared between Pol III and Pol I are shown in orange; and subunits unique to Pol III are shown in blue. Diseases associated with mutations in the corresponding genes are indicated. The leukodystrophy-causing mutations generally lead to deficiency in Pol III activity and altered levels of its transcription products such as BC200 and some tRNAs. Some mutations in Pol III subunits impair cytoplasmic Pol III function leading to deficient immunity against severe VZV (varicella-zoster virus) infections. Immune response to altered *POLR3A* locus in cancer tissues can cause scleroderma as a paraneoplastic autoimmune disease.

**Figure 2.**
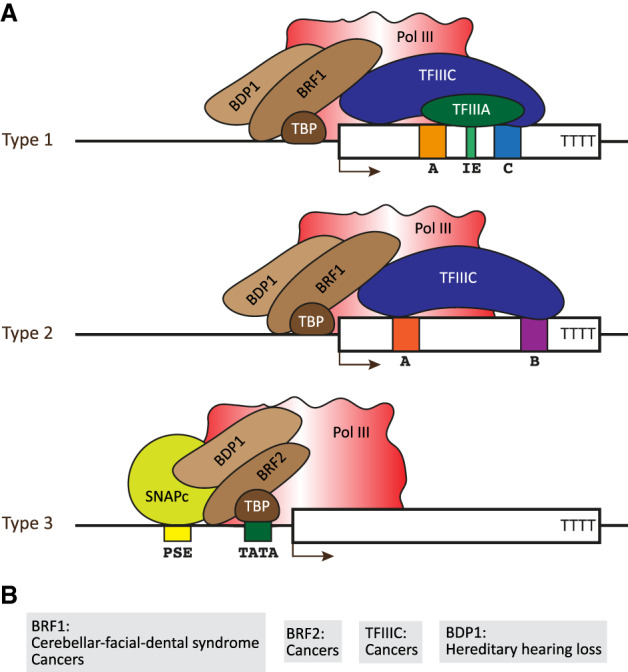
Pol III transcription machinery and associated diseases. (*A*) The structures of type 1, 2, and 3 Pol III promoters and their associated transcription factors are shown. For BRF1-TFIIIB (type 1 and type 2 promoters) and BRF2–TFIIIB (type 3 promoters), the subunit composition is indicated (brown). (*B*) Diseases associated with mutations in the genes coding for the indicated transcription factors and their deregulation. Mutations in *BRF1* are detected in patients with cerebellar-facial-dental syndrome. These mutations were shown to impair Pol III activity and to cause growth defects in yeast. In cancers, both increased (hepatocellular carcinoma [HCC] and breast cancer) and decreased (kidney and colorectal tumors) levels of BRF1 have been shown, suggesting positive as well as negative roles in tumorigenesis. BRF2 levels have been shown to increase in several cancers including melanoma, gastric, kidney, esophageal, and lung cancers. High *BRF2* expression in cancers can lead to increased production of SeCys (selenocysteine) tRNA and thus selenoproteins, which increases the tumor cells’ resistance to oxidative stress and apoptosis. Increased levels of TFIIIC subunits in ovarian cancer lead to increased Pol III transcription.

Pol III transcription is regulated in different biological processes such as the cell cycle (White et al. 1995), differentiation (Alla and Cairns 2014; Van Bortle et al. 2017), development (Schmitt et al. 2014), regeneration (Yeganeh et al. 2019), and cellular stress (Upadhya et al. 2002). Its activity is controlled by MAF1, a protein conserved from yeast to human that binds to the enzyme and represses its activity, and by a number of tumor suppressors and oncogene products that act on Pol III transcription factors (for review, see Marshall and White 2008; Gjidoda and Henry 2013). Knocking out *Maf1* in mice results in animals that are lean, obesity-resistant, and metabolically inefficient, and that display increased Pol III occupancy at Pol III genes (Bonhoure et al. 2015). However, so far, no links have been uncovered between defects in MAF1 regulation or function and human disorders. In contrast, deregulation of Pol III activity and deregulation of the expression of some Pol III genes caused by tumor suppressor or oncogene deregulation are observed in many cancers and contribute to tumorigenicity. Moreover, diminished Pol III activity due mutations within the enzyme causes leukodystrophies and related disorders as well as heightened susceptibility to severe complications from varicella-zoster infections. Defects in one specific Pol III product can also have dire consequences, as in the example of RMRP, whose reduced expression or mutation can cause cartilage hair hypoplasia (CHH).

## Pol III transcription apparatus

Various disorders have been linked to mutations in components of the Pol III transcription apparatus, including subunits of the enzyme itself (Fig. 1) and of its associated transcription factors (Fig. 2). For example, a homozygous missense mutation in *POLR3H* was shown to cause primary ovarian insufficiency in humans and delayed pubertal maturation and decreased fertility in mice (Franca et al. 2019). A linkage and exome sequencing study identified a homozygous mutation in *BDP1* associated with hereditary hearing loss (Girotto et al. 2013). In many cases, the mechanisms linking the observed mutations and the phenotypes are not understood beyond the mutations leading to lower activity of Pol III transcription, either as a result of poorer enzyme assembly or poorer recruitment to Pol III promoters. As can be expected for a ubiquitously expressed and essential transcription machinery, none of the mutations cause a complete loss of function.

### Pol III subunits and Pol III-related leukodystrophies

Hypomyelinating leukodystrophies constitute a group of heterogeneous, inherited neurodegenerative diseases with overlapping clinical phenotypes characterized by neurological (cerebellar, extrapyramidal, pyramidal, and cognitive) and nonneurological (dental, endocrine, and ocular) abnormalities. The diseases include hypomyelination, hypodontia, and hypogonadotropic hypogonadism (4H) syndrome; ataxia, delayed dentition, and hypomyelination (ADDH); tremor ataxia with central hypomyelination (TACH); leukodystrophy with oligodontia (LO); hypomyelination with cerebella atrophy and hypoplasia of the corpus callosum (HCAHC); and Wiedemann-Rautenstrauch syndrome (see Bernard and Vanderver 2012 and references therein). Remarkably, the identification of the genetic mutations involved has revealed that all these disorders result from mutations in genes encoding Pol III subunits and can thus be described as being part of a spectrum of Pol III-related leukodystrophies.

Most of the mutations are homozygous or compound heterozygous mutations in subunits POLR3A and POLR3B (41% and 49%, respectively) (Bernard and Vanderver 2012), although mutations in POLR1C, POLR3K, and POLR3GL have also been reported (Fig. 1; Bernard et al. 2011; Daoud et al. 2013; Thiffault et al. 2015; Dorboz et al. 2018; Terhal et al. 2020). Mutations predicted or shown to lead to deficient splicing or premature termination, and thus to a nonfunctional protein, are coupled with alleles carrying point mutations in highly conserved positions. The mutations lead to lower amounts and/or various, sometimes poorly understood, defects in the enzyme.

An interesting case is that of the p.N32I and p.N74S mutations in POLR1C, a subunit shared between Pol I and Pol III. Pull-down experiments from extracts of HeLa cells expressing FLAG-tagged cDNA versions of wild-type POLR1C or POLR1C carrying either of these mutations revealed equivalent protein expression levels but impaired Pol III, but not Pol I, assembly, as well as impaired Pol III nuclear import (Thiffault et al. 2015). Chromatin immunoprecipitation sequencing (ChIP-seq) experiments showed decreased POLR1C binding to all types of Pol III, but not Pol I, genes. In contrast, the POLR1C carrying the p.R279Q mutation, associated with Treacher Collins syndrome, affected neither Pol III nor Pol I assembly, but impaired POLR1C targeting to the nucleolus, where Pol I acts. Thus, different mutations in a subunit common to Pol I and Pol III differentially affects Pol I and Pol III activities, leading to different disorders (Thiffault et al. 2015).

Although it is clear that mutations in Pol III subunits can lead to decreased enzyme activity, the exact consequences of this decreased activity on various Pol III products have been difficult to ascertain, in large part because of the difficulty of precisely quantifying RNAs that can be highly structured and modified. Nevertheless, in most cases tested, such mutations result in lower amounts of Pol III products, although which RNAs are affected varies. Furthermore, and as can be expected, changes in levels of mRNAs and proteins can be observed, which could result from a large number of direct or indirect effects such as changes in translation due to altered 5S rRNA (ribosomal RNA) or tRNA levels, altered levels of RNAs involved in the processing of other RNAs (e.g., RPPH1 and RMRP), or altered levels of regulatory RNAs (e.g., 7SK). For example, small RNA sequencing (ultra-high-throughput RNA sequencing, which can be used to quantify unmodified pre-tRNAs as well as some other Pol III products) of RNA extracted from the blood of healthy controls and leukodystrophy patients with c.1771-6C > G mutation in *POLR3A*, revealed a general but mild decrease in levels of tRNA precursors and 7SL RNA together with an increase in levels of 5S, 7SK, RMRP, and RPPH1 RNAs. Furthermore, the levels of some mRNAs encoding factors involved in splicing and proteostasis were altered (Azmanov et al. 2016). In skin fibroblasts of hypomyelinating leukodystrophy patients with a biallelic pR41W mutation in POLR3K predicted to disrupt its interaction with POLR3B, no changes in the level of the few tRNAs tested by RT-qPCR were observed, with the notable exception of tRNA_i_^Met^ (initiator methionine tRNA), which showed a mild decrease. In contrast, the level of 5S rRNA and 7SL RNA were strongly reduced (Dorboz et al. 2018).

In another study focused on the leukodystrophy-associated p.M825V mutation in POLR3A, HEK293 cells were engineered with CRISPR/Cas9 to be compound heterozygous with the M852V mutation on one allele and an indel causing a frameshift and premature stop codon on the other, and thus expressing only p.M825V POLR3A. Small and standard RNA sequencing revealed a general trend to decreased Pol III transcript levels, an effect that was more pronounced for type 2 Pol III genes, specifically for some pre-tRNAs, BC200, and 7SL RNA. Among these RNAs, BC200 was of special interest because it is highly expressed in the central nervous system (CNS) and was first characterized as a translation modulator in neuronal cells. BC200 was also decreased in patient fibroblasts and in an oligodendroglial cell line engineered similarly to the HEK293 cells to express only p.M825V POLR3A (Choquet et al. 2019a). Furthermore, comparison of the transcriptomes and proteomes of the p.M825V and a BC200 KO oligoendroglial cell line revealed partially overlapping changes, with the effects generally larger in the BC200 KO cells. Perhaps most relevant to the disease, after differentiation into more mature oligodendrocytes, *MBP* (myelin basic protein) mRNA levels, encoding one of the major myelin proteins in the central nervous system, were decreased, although less so in BC200 KO than in p.M825V cells. Thus, part of the *POLR3A*-mutant phenotype may be due to decreased BC200 levels (Choquet et al. 2019a).

Obtaining mouse models of Pol III-related leukodystrophy has proven challenging. Mice carrying a homozygous p.G672E POLR3A mutation mediating leukodystrophy in humans did not show any detectable neurological abnormality nor any changes in Pol III transcript levels in the brain. Furthermore, the same mutation also had no clear effect on Pol III assembly, localization, and occupancy of a few tested target genes in human cell lines (Choquet et al. 2017), and the effect of this mutation on Pol III transcript levels in patients is not known. It would be valuable to compare systematically its effect on the human and mouse Pol III and Pol II transcriptome as well as proteome, as this may pinpoint factors causal for the disease. For example, are levels of BC200 affected in patients? Indeed, although BC1, whose levels were not affected in the mouse model, is considered the functional homolog of the primate specific BC200, the two genes have different evolutionary origins and are likely to differ in their cellular functions. A second mouse model carrying the POLR3B leukodystrophy-mediating mutation p.R103H, which led to impaired Pol III assembly in human cultured cell lines, was embryonic lethal in homozygotes (Choquet et al. 2019b).

These studies suggest that a Pol III subunit mutation can have specific effects on transcription of some Pol III genes, in line with the many studies documenting differences in the Pol III transcriptome of different cell types and contrasted responses of different Pol III genes to changes in cellular environment (Schmitt et al. 2014; Orioli et al. 2016; Van Bortle et al. 2017; Yeganeh et al. 2019). These varied responses probably underline the varied phenotypes of Pol III-related leukodystrophy. BC200, which is also deregulated in neurodegeneration (see BC200 section below) and tRNAs, which display a processing deficit in neurological disorders (Schaffer et al. 2019) are promising candidates to be major players in the disease.

### Pol III transcription factor subunits: growth failure and central nervous system anomalies

Several of the TFIIIB and TFIIIC subunits (Fig. 2) have been found mutated or deregulated in a number of disorders, among them BRF1, BRF2, BDP1, and TFIIIC2 subunits. Mutations in *BRF1*, like those in Pol III subunits described above, are involved in neurological diseases. Missense compound heterozygous or homozygous *BRF1* mutations were detected in patients with cerebellar–facial–dental syndrome, a newly described syndrome manifested by intellectual disability, cerebellar hypoplasia, short stature, and dental anomalies (Borck et al. 2015). When introduced in yeast *BRF1*, some of the mutations led to growth defects, decreased Brf1 occupancy at tRNA genes and impaired Pol III transcriptional activity. Experiments performed in zebrafish suggest that the mutations are causal. Zebrafish have two *brf1* genes, only one of which (*brf1b)* is greatly up-regulated during development. Down-regulation of *brf1b* led to reduction of head size and cerebellar hypoplasia. Rescue experiments were performed with human *BRF1* sequences coding either for a shorter human BRF1 isoform lacking the zinc-binding domain (McCulloch et al. 2000) or for the full-length protein. In the first case, all of the mutations seen in patients were deleterious, albeit to varying degrees, whereas in the second, some were benign, suggesting an isoform-specific effect (Borck et al. 2015). It should be noted that the relative levels of human BRF1 protein isoforms in different cell types and their specific functional roles for Pol III transcription have not been characterized. It will be important to examine these isoforms in the central nervous system.

Heterozygous *BRF1* mutations, including a frameshift and a missense mutation (p.P292R) that affects the same amino acid (p.P292) as one of the mutations (p.P292H) described by Borck et al. (2015) (Jee et al. 2017), were also uncovered in another family with growth failure and central nervous system anomalies. The P292H as well as a P292R mutations were further shown to prevent yeast growth when introduced into yeast Brf1 (Jee et al. 2017), again consistent with decreased BRF1 activity causing or contributing to the disorder.

### Pol III transcription apparatus and cancer

Pol III transcription is up-regulated with cell growth and cell proliferation (Goodfellow and White 2007; Dumay-Odelot et al. 2010), and TFIIIB is a target of both tumor suppressors and oncoproteins. This raises the possibility of cancer-associated deregulation of TFIIIB subunits and subunits of other Pol III transcription factors. Indeed, the levels of some TFIIIC and Pol III subunits, as well as those of all TFIIIB subunits (BRF1, BRF2, TBP, and BDP1) increased when primary human IMR90 fibroblasts were transformed with successive defined genetic steps, namely ectopic expression of (1) HPV16 proteins E6 and E7, leading to inactivation of RB1 and TP53, respectively, (2) SV40 small t antigen, leading to inactivation of protein phosphatase 2A (PP2A), (3) constitutively active RAS (RAS-G12V), and (4) the catalytic component of telomerase (TERT [telomerase reverse transcriptase]) (Durrieu-Gaillard et al. 2018). Increased levels of TFIIIC subunits were also observed in ovarian tumors, together with increased levels of pre-tRNA, 5S rRNA and 7SL RNA. Addition of TFIIIC subunits to ovarian cell extracts increased Pol III transcription, suggesting that TFIIIC levels are limiting in these cells. The increase in TFIIIC levels did not appear to simply reflect cell proliferation, as serum deprivation decreased tRNA levels, but not levels of TFIIIC subunits (Winter et al. 2000).

Deregulation of TFIIIB subunits has been repeatedly observed in cancer cells. Increased levels of BRF1 were observed in hepatocellular carcinoma (HCC) tissues and were associated with poor survival (Zhong et al. 2016). Alcohol consumption, which is known to increase the risk of HCC, was linked to higher BRF1 levels in both normal liver and HCC tissues. In cellular models, Pol III activation by ethanol a well as cellular transformation by EGF and ethanol were reduced in *Brf1* knockdown cells (Zhong et al. 2016).

In breast cancer tissues, increased levels of BRF1 were associated with high ER (estrogen receptor) levels; ERα was then found to associate with the *BRF1* promoter and increase its expression in breast cancer cell lines, and to interact with BRF1. Consistent with this last observation, ERα could be localized to a tRNA^Leu^ and 5S rRNA genes by ChIP-qPCR, and transcription from these genes was decreased upon knockdown of either ERα or *BRF1*. Treatment of the MCF7 breast cancer cell line with the ER antagonist tamoxifen decreased (1) *BRF1* mRNA and protein levels, (2) transcription from the tRNA^Leu^ and 5S rRNA genes, and (3) colony formation induced by ethanol. Thus, hormone therapy may act in part by decreasing BRF1 levels (Fang et al. 2017). In yet another system, anchorage-independent growth and tumorigenesis of Rat1a fibroblasts mediated by TBP or c-MYC overexpression was inhibited by *Brf1* knockdown (Johnson et al. 2008). These results are all consistent with increased BRF1 levels and activity contributing to the transformation process and to tumorigenesis. However, there are some observations that suggest that BRF1 is a tumor suppressor rather than a protein whose increased expression favors cancer.

Supporting the role of Brf1 as a tumor suppressor, knockdown of *Brf1* expression with antisense RNA in MEFs (mouse embryonic fibroblasts) led to partial bypass of senescence, reduced p53 transcriptional activity and p16-induced cell cycle arrest, and enhanced ras-mediated transformation. The *BRF1* gene is located in a chromosomal region showing significant allelic loss in different tumors, and indeed, decreased *BRF1* mRNA levels were observed in 50% of the kidney tumors and 23% of the rectal tumors investigated (Leal et al. 2008). In another study, heterozygous mutations in *BRF1* were observed in several patients with familial colorectal cancer. These mutations reduced protein levels as determined by expression of the mutants in a colorectal cancer cell line or led to nonfunctional proteins as determined by the inability of yeast cells carrying corresponding mutations in yeast *BRF1* to grow (Bellido et al. 2018). It is important to note, however, that in these studies, the effect of the tumor *BRF1* mutations on Pol III activity and on synthesis of various Pol III transcripts was not examined. It is possible that in the cellular environment of these particular tumors, reduced synthesis of one or several Pol III products contributes more to tumorigenesis than the general increase in Pol III transcription linked to *BRF1* overexpression observed in other tumors. It is also possible that Pol III transcription remains, in fact, high in these tumors due to a secondary mutation. Finally, the mutated BRF1 proteins may have acquired a novel function that contributes to tumorigenesis.

BRF2 is part of the BRF2–TFIIIB complex, which is involved in recruitment of Pol III to genes with type 3 promoters. Like that of *BRF1*, expression of *BRF2* was increased when primary human IMR90 fibroblasts were transformed with successive defined genetic steps (Durrieu-Gaillard et al. 2018), and *BRF2* expression was found increased in Oncomine data sets from melanoma as well as gastric and kidney cancers (Cabarcas and Schramm 2011). Increased *BRF2* expression was also detected in esophageal squamous cell cancer tissues (Lu et al. 2013) and in nonsmall cell lung carcinomas, where it was associated with higher intratumoral microvessel density, a marker of tumor angiogenesis (Lu et al. 2014), and lower E-Cadherin and higher N-Cadherin levels, a marker for epithelial to mesenchymal transition (Tian et al. 2014). In both cases, *BRF2* overexpression was also associated with poor patient prognosis. Nonsmall cell lung carcinoma can be divided into two subtypes, lung squamous cell carcinoma (SqCC) and adenocarcinoma (AC). In a study analyzing the two subtypes separately, *BRF2* overexpression associated with gene amplification was found in SqCC (40% of tumors) and preinvasive SqCC lesions, but not in AC lesions. *BRF2* knockdown in a lung SqCC cell line decreased cell proliferation and colony formation, and overexpression in a nontransformed lung epithelial cell line increased cell proliferation. Tumors with the highest *BRF2* expression showed 86 genes differentially expressed, with functions in posttranscriptional modification, gene expression, and cancer. These studies establish *BRF2* as a lineage-specific oncogene characteristic of SqCC, but not adenocarcinoma, lesions (Lockwood et al. 2010).

An interesting difference between BRF2 and BRF1 is the presence, in BRF2, of a redox-sensing module based on oxidative modification of cysteine C361. Treatment of lung fibroblast cells with an oxidative stress-inducing reagent led to decreased levels of BRF2-dependent Pol III transcripts, notably SeCys pre-tRNA, to a decreased level of selenoproteins involved in oxidative stress response, and ultimately to apoptosis. Overexpression of *BRF2* in a mammary epithelium cell line with low endogenous BRF2 levels increased selenoprotein levels and led to resistance to oxidative stress. These results suggest that increased levels of BRF2 in cancer cells ensures abundant production of SeCys tRNA and thus of selenoproteins, which in turn confers resistance to oxidative stress and apoptosis (Gouge et al. 2015). A comparison of SeCys pre-tRNA and selenoprotein levels in tumors overexpressing or not overexpressing *BRF2*, for example SqCC and AC tumors, remains to be done.

### Cytoplasmic Pol III and varicella-zoster infections

Unlike Pol I and Pol II, which are thought to be entirely nuclear, Pol III has been shown to have both nuclear and cytoplasmic functions. In the cytoplasm, Pol III acts as a cytosolic DNA sensor that recognizes and transcribes, in a promoter-independent manner, AT-rich DNA from invading pathogens into RNA intermediates containing a 5′ triphosphate group (5′-ppp RNA). 5′-ppp RNA serves then as a ligand for RIG-I-like receptors to trigger an innate immune response and production of type I interferons (IFNs) (Ablasser et al. 2009; Chiu et al. 2009). Cytoplasmic Pol III plays an important role in preventing severe varicella-zoster virus (VZV) infections. VZV, which possesses several very AT-rich regions (70%–80% AT) in its genome, can cause varicella (chickenpox) upon primary infection and herpes zoster (shingles) following reactivation. However, in rare cases, severe complications can evolve from both primary infections and reactivation. Whole exome sequencing of DNA from patients suffering from severe VZV infection in the CNS or the lungs identified heterozygous missense mutations in *POLR3A*, *POLR3C,* or both (Ogunjimi et al. 2017; for review, see Carter-Timofte et al. 2018b). Peripheral blood mononuclear cells (PBMC) from patients contained similar levels of POLR3A, POLR3C, and 5S rRNA compared with healthy controls. Nevertheless, these cells displayed only weak IFN induction upon stimulation with poly(dA:dT) and were unable to control VZV infection, a phenotype that was rescued upon reintroduction of wild-type *POLR3A* and *POLR3C* genes by lentiviral transduction (Ogunjimi et al. 2017). Further studies uncovered mutations in *POLR3A*, *POLR3E*, and *POLR3F* in patients with VZV reactivation-caused CNS infection and vasculitis. PBMCs of these patients again showed impaired antiviral response and increased VZV replication after infection (Carter-Timofte et al. 2018a; Carter-Timofte et al. 2019). The available data thus identify an important role for cytoplasmic Pol III transcription in triggering IFN induction, and yet many fundamental questions regarding the mechanisms involved have not yet been addressed. Thus, is cytoplasmic Pol III identical to nuclear Pol III or does it differ slightly in subunit composition? Transcription of AT-rich DNA is promoter-independent, but what are the exact requirements in terms of template? And does it require some of the known Pol III transcription factors? Does it require other factors? An answer to these questions will not only go a long way toward explaining the role of Pol III in innate immunity, it might also lead to the discovery of yet unsuspected Pol III functions.

### Mutations in Pol III subunits, cancer, and scleroderma

Systemic sclerosis or scleroderma is an autoimmune rheumatic disease characterized mainly by fibrosis and vascular damages affecting skin and internal organs. Patients with scleroderma are in general at higher risk of developing cancer than the general population, but in patients with anti-Pol III antibodies in their serum, cancer and scleroderma development often occur nearly concurrently. Furthermore, analysis of the *POLR3A* locus in tumors from such patients revealed frequent (six of eight patients) somatic mutations (Joseph et al. 2014). This observation suggests that mutations occurring in the *POLR3A* locus during tumor growth lead to an immune response directed against both cancer cells carrying the mutated *POLR3A* locus as well as to normal cells expressing only wild-type *POLR3A*, and thus to an autoimmune response. Thus, in these cases, scleroderma might correspond to a paraneoplastic disease (Shah and Casciola-Rosen 2015). This hypothesis would be greatly strengthened by the detection, in cases of scleroderma with anti Pol III antibodies but no alterations at the *POLR3A* locus, of mutations in loci encoding other Pol III subunits (Shah and Casciola-Rosen 2015).

## Pol III products

Pol III synthesizes a wide variety of short RNAs with different functions. Not surprisingly, the levels of these products are altered in various disorders, especially in different cancers, not necessarily as a result of mutations affecting the basal Pol III transcriptional machinery or Pol III regulators, but also as a result of genomic rearrangements or other mutations directly affecting certain target loci. At a very general level, it seems obvious that increased levels of many tRNAs and 5S rRNA are required to allow the increased proliferation and translation rate of many cancer cells. However, how deregulation of a particular Pol III product contributes to a disorder is in most cases not or only very partially understood. Just as an example, levels of U6 snRNA were found elevated in cancer cells (Lockwood et al. 2010) and in serum of breast cancer patients with metastasis, but whether this affected mRNA splicing or some other process is unknown (Appaiah et al. 2011). Similarly, Y RNAs, which have been reported to be required for chromosomal DNA replication (Kowalski and Krude 2015), have been found both elevated and reduced in various types of cancer, but the processes affected by these changes are unknown (Christov et al. 2008; Nientiedt et al. 2018; Tolkach et al. 2018). Below, we review examples of deregulated Pol III products in diseases for which we have some insight as to the mechanisms involved.

## tRNAs

Altered levels of tRNAs have been observed repeatedly in cancer cells and in a few cases, specific overexpressed tRNAs have been implicated in specific cancer-contributing mechanisms. In addition, some tRNA mutations have been shown to lead to neurodegeneration.

### Altered tRNA levels and cancer

**Figure 3.**
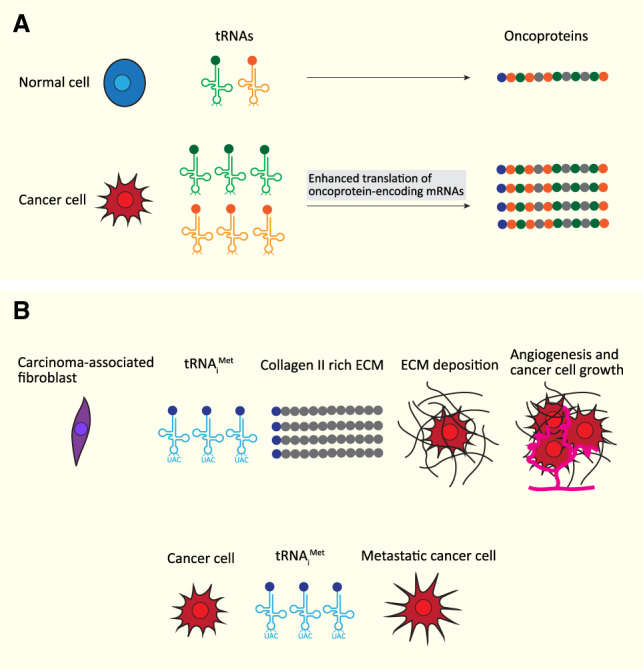
tRNA overexpression in cancer. (*A*) Overexpression of tRNA isoacceptors whose corresponding codons are enriched in oncoprotein-encoding mRNAs are observed in breast cancer (Pavon-Eternod et al. 2009; Goodarzi et al. 2016). (*B*, *top* panel) Overexpression of tRNA_i_^Met^ in carcinoma-associated fibroblasts enhances translation of secreted collagen II-enriched ECM components, leading to angiogenesis and cancer cell growth (melanoma and lung carcinoma) (Clarke et al. 2016). (*Bottom* panel) Overexpression of tRNA_i_^Met^ by the melanoma cells leads to increased migration and invasion, which is dependent on α5β1 integrin (Birch et al. 2016).

Genome-wide profiling of tRNAs in breast tumors and normal breast tissues revealed up-regulation of several tRNAs to be associated with poor patient prognosis, raising the possibility of a causal effect (Krishnan et al. 2016). Indeed, several studies confirm a role for tRNA deregulation in tumor progression. Thus, global levels of tRNAs measured by tRNA microarrays was shown to increase in breast tumors compared with normal breast tissues, and in breast cancer cell lines compared with noncancer breast epithelial cell lines with comparable proliferation rates. Overexpression varied among different tRNA isoacceptor and in breast cancer cell lines, there was a positive association between tRNA overexpression and codon usage of overrepresented codons in cancer-related genes, which may favor their translational efficiency (Pavon-Eternod et al. 2009). Furthermore, tRNA profiling in highly metastatic breast cancer cell lines and metastatic breast tumors revealed alterations in certain tRNA levels, with tRNA^Arg^_CCG_ and tRNA^Glu^_UUC_ being two highly up-regulated isoacceptors. Pre-tRNAs levels and copy number of the corresponding genes were also elevated. Knockdown of either tRNA decreased the invasion and lung colonization capacity of breast cancer cell lines, whereas their overexpression had the opposite effect. Ribosome profiling in cells overexpressing either of the two tRNAs showed significantly higher ribosome occupancy of mRNAs with higher matching codon content, which led to increased mRNA stability and corresponding protein levels. In tRNA^Glu^_UUC_ overexpressing cells, EXOSC2 and GRIPAP1 protein levels were increased, and knocking down the expression of both these genes in mice inhibited tRNA^Glu^_UUC_ overexpression-mediated enhanced metastasis and invasion. This study indicates that deregulation of tRNA levels can contribute to metastasis by enhancing translation of specific mRNAs (Fig. 3; Goodarzi et al. 2016). This is consistent with previous observations showing that specific sets of tRNAs are induced in proliferation or differentiation to adjust to the translational program depending on the cell state (Gingold et al. 2014).

Another example is that of tRNA_i_^Met^, which was shown to be elevated in carcinoma-associated fibroblasts as compared with normal fibroblasts. Carcinoma-associated fibroblasts deposit the extracellular matrix (ECM), which supports angiogenesis and tumor growth. Accordingly, mice with an additional copy of RNA_i_^Met^ gene displayed increased angiogenesis and growth of subcutaneous melanoma or lung carcinoma cell allografts. Furthermore, upon overexpression of tRNA_i_^Met^ in immortalized MEFs, increased levels of many secreted ECM components were observed, in particular that of collagen II. This likely resulted from increased collagen II translation, as polysomal content on collagen II mRNA was specifically increased even though general protein synthesis was unaltered. Furthermore, endothelial cell migration was increased by ECM from fibroblasts overexpressing RNA_i_^Met^ but this effect was suppressed when collagen II expression was knocked down. Similarly, the increased tumor vascularity and growth in mice with additional copy of RNA_i_^Met^ gene was specifically suppressed upon administration of an inhibitor of collagen processing (Fig. 3; Clarke et al. 2016).

Unlike overexpression of tRNA_i_^Met^ in stromal cells such as carcinoma-associated fibroblasts, overexpression in tumor cells lead to the acquisition, in a cell autonomous manner, of migratory and invasive characteristics (Birch et al. 2016). Furthermore, the effect was dependent on integrin as knockdown of the α5 subunit of α5β1 integrin reversed tRNA_i_^Met^ overexpression effect (Fig. 3). Thus, tRNA_i_^Met^ overexpression can promote cancer in at least two ways: overexpression in stromal cells induces alterations in the composition of the deposited ECM, which in turn increases endothelial cell mobility to promote tumor angiogenesis and growth. On the other hand, overexpression in tumor cells increases, in an α5β1 integrin-dependent manner, their migration and invasion capacity. Importantly, it did not increase cell proliferation, thus excluding a very general effect in which higher translation initiation rates mediated by tRNA_i_^Met^ overexpression would lead to enhanced protein synthesis to support higher proliferation rates (Birch et al. 2016).

In addition to deregulation of tRNA gene expression, mechanisms involving tRNA-derived fragments, which can function either as oncogenic promoters or tumor suppressors depending on the type of cancer, have been described. These have been recently reviewed and are not described here (see Sun et al. 2018 and references therein).

### tRNA mutations and neurodegeneration

Several mutations in mitochondrial tRNA genes have been described that impair mitochondrial function leading to diseases such as mitochondrial myopathy with diabetes or Leigh syndrome, a neurological disorder (Schaffer et al. 2019). Unlike for mitochondrial tRNA genes, there is only one identified example of disease-causing mutation in a nuclear-encoded tRNA, which was identified in a *N-*ethyl*-N*-nitrosourea mutagenesis screen for neurological phenotypes in mice (Ishimura et al. 2014). The point mutation occurs in the tRNA ^Arg^_UCU_-encoding tRNA gene *n-Tr20*, which is expressed specifically in the CNS. The mutation appears to change the length of the primary transcript and affect transcript processing, leading to accumulation of a pre-tRNA that is shorter than WT pre-tRNA, reduction of the tRNA ^Arg^_UCU_ pool, and reduction of aminoacetylation. In combination with a mutation affecting a consensus splice donor site in *Gtpbp2* resulting in the loss of GTPBP2, a factor involved in alleviating ribosomal stalling, the mutation leads to severe neurodegeneration in mice. In *n-Tr20* mutant mice, mild ribosome pausing was observed at the tRNA ^Arg^_UCU_ cognate AGA codons, which was much increased in the absence of GTPBP2. Thus, a decrease in functional tRNA ^Arg^_UCU_ leads to ribosome pausing, which can be alleviated in the presence, but not in the absence, of GTPBP2 (Ishimura et al. 2014).

## 7SL and Alu RNA

The 7SL RNA genes encode 7SL RNA, which, as part of the signal recognition particle, has an important role in polypeptide translocation to the endoplasmic reticulum (Walter and Blobel 1982). Apart from the 7SL genes, the human genome contains about 1 million copies (11% of human genome) of Alu elements, which are derived from the 7SL gene through a process of retrotransposition and contain internal Pol III promoters (Ullu and Tschudi 1984; Deininger 2011). However, under normal circumstances, most of these Alu sequences are not expressed (Deininger 2011; Kramerov and Vassetzky 2011).

7SL RNA levels were found to be greatly increased in different cancer tissues. Moreover, 7SL knockdown decreased cell proliferation of various cell lines and led to cell cycle arrest, inhibition of DNA replication, cellular senescence, and autophagy in HeLa cells, suggesting some 7SL RNA function favoring cell proliferation. Indeed, 7SL RNA was found to interact with the 3′ UTR (untranslated region) of *TP53* mRNA and suppress its translation. 7SL knockdown led to enhanced HuR protein binding to the *TP53* mRNA 3′ UTR, increased translation, and increased TP53 protein levels, suggesting that 7SL RNA and HuR compete for binding to the *TP53* mRNA 3′ UTR (Abdelmohsen et al. 2014). Thus, in addition to its canonical role in protein translocation, 7SL RNA inhibits *TP53* translation and thus its proliferation-suppressive function.

**Figure 4.**
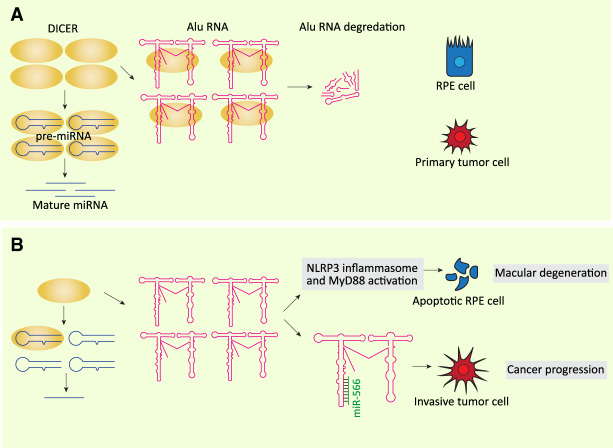
Alu RNA in diseases. (*A*) DICER is not only involved in miRNA maturation, but also degrades Alu RNA. (*B*) When DICER is down-regulated, increased levels of Alu RNA leads to cancer progression (Di Ruocco et al. 2018) and macular degeneration (Kaneko et al. 2011; Tarallo et al. 2012) through different mechanisms that are independent from miRNA deficiency. In RPE cells, Alu overexpression activates the NLRP3 inflammasome and MyD88, leading to cell apoptosis, whereas in colorectal cancer cells Alu RNA sequesters miR-566 leading to increased invasion capacity of the cells.

As for 7SL RNA, elevated Alu RNA levels have been reported in a number of cancer cells; for example, in hepatocellular carcinoma tissues (Tang et al. 2005). Examination of two colorectal cancer cell lines representing primary carcinoma or metastatic stages of the disease revealed decreased DICER1 and increased Alu RNA with cancer progression (Di Ruocco et al. 2018), consistent with Alu RNA being degraded by DICER1 (Kaneko et al. 2011). Transfection of Alu RNA into the primary carcinoma cell line induced epithelial to mesenchymal transition and increased migration and invasion. The same effect could be obtained with DICER1 knockdown, and this was inhibited by simultaneous Alu RNA knockdown. Simultaneous Alu RNA knockdown did not rescue the miRNA (microRNA) deficit resulting from DICER1 knockdown, indicating a miRNA-independent function of DICER1 through Alu RNA. This function could be shown to consist of capturing miR-566, as (1) increased Alu RNA was accompanied by decreased miR-566 levels, and (2) transfection of miR-566 inhibited Alu-induced epithelial to mesenchymal transition (Fig. 4; Di Ruocco et al. 2018).

DICER-mediated regulation of Alu RNA plays a role in another disease. Decreased levels of DICER1 accompanied by a large increase in Alu RNA was observed in retinal pigmented epithelium (RPE) of human eyes with geographic atrophy (GA), an advanced form of age-related macular degeneration (Kaneko et al. 2011). Depletion of DICER1 in human RPE cells induced accumulation of Alu RNA and cell death, which was prevented by Pol III inhibition. Furthermore, DICER1 depletion-mediated cell death could be prevented by Alu RNA knockdown despite persistent miRNA deficit, suggesting that Alu RNA accumulation, and not miRNA deficit, plays a role in macular degeneration (Kaneko et al. 2011). In a follow-up report, the effects of Alu RNA accumulation in RPE of wild-type mice or mice lacking various players of the innate immune response as well as in various mouse and human cell lines were examined. Alu RNA accumulation in mouse RPE led to cell death and degeneration, an effect not seen in *MyD88*^−/−^ mice or when MyD88 was concomitantly inactivated. Further experiments revealed that Alu RNA activated the generation of mitochondrial reactive oxygen species (ROS), leading to an activation cascade of successive substrates, namely the NLRP3 inflammasome, caspase I, IL-18, and MyD88, and, ultimately, RPE cell death and degeneration (Fig. 4; Tarallo et al. 2012). These results indicate that deregulated levels of Alu RNAs can be highly deleterious to the cell, and that in addition to transcription repression of Alu loci, there is another mechanism, involving DICER1-mediated degradation, to keep Alu levels low.

## RPPH1

Ribonuclease P RNA component H1 (RPPH1) is involved in the processing of the 5′ end of pre-tRNAs. In addition, however, various studies have implicated RPPH1 in diseases through other mechanisms. Thus, RPPH1 levels were found increased in breast cancer tissues and cell lines. RPPH1 overexpression in breast cancer cell lines increased proliferation and colony formation, whereas its knockdown had opposite effects and led to decreased tumor size in nude mice. An inverse correlation between RPPH1 and miR-122 levels in breast cancer tissues and cell lines was observed, and miR-122 overexpression decreased cell proliferation mediated by RPPH1 overexpression. RPPH1 may thus alter miR-122 levels and ultimately the levels of miR-122 targets (Zhang and Tang 2017).

Rpph1 was also up-regulated in renal tissue of mice with diabetic nephropathy; in fact it was the most up-regulated long noncoding RNA. In cultured mesangial cells, it promoted cell proliferation and expression of inflammatory cytokines. Rpph1 was shown to bind Gal-3, a biomarker of diabetic nephropathy, which itself was increased in diseased renal tissue and enhanced mesangial cells proliferation. Further experiments suggested that Rpph1 somehow enhances the expression of Gal-3, which itself was shown to play a role in the regulation of inflammation and proliferation of mesangial cells via the Mek/Erk pathway (Zhang et al. 2019).

## New Pol III transcription units with type 3 promoters

Putative transcription units with type 3 Pol III promoters, located in antisense orientation in the intron of genes with neuronal functions, have been examined for a possible role of their transcripts in Alzheimer's disease. We reported previously the presence of a short interspersed nuclear element (SINE) with a type 2 Pol III promoter located in an antisense orientation within the first intron of the *Polr3e* gene, whose Pol III occupancy interfered in *cis* with Pol II elongation (Yeganeh et al. 2017). Unlike the SINE, these type 3 Pol III transcription units—namely, 17A, 38A, NDM29, 51A, and 45A, located within introns of the *GPR51*, *KCNIP4*, *ASCL3*, *SORL1*, and *APBB2* genes, respectively—act through the encoded RNA in *trans*, generally to promote alternative splicing (Massone et al. 2011a,b, 2012; Ciarlo et al. 2013; Penna et al. 2013). Overexpression of 17A, 38A, NDM29, and 51A in neuroblastoma cell lines increased secretion of amyloid β (Aβ) as a result of enhanced amyloid precursor protein (APP) processing and sometimes also increased protein levels. Consistent with this observation, elevated levels of these Pol III transcripts were observed in postmortem brain samples of Alzheimer's disease patients. In neuroblastoma cell lines, the expression of 17A, 38A, and NDM29 was up-regulated by inflammatory stimuli. In contrast, overexpression of the 45A transcription unit in HEK293 cells led to decreased APP processing and Aβ secretion, yet also to an increase in the Aβ-x42/Aβ-x40 ratio, which is favorable for Aβ aggregation and constitutes a biomarker of Alzheimer's disease. Thus, deregulation of a number of intron-located Pol III transcription units, in some cases by inflammatory signals, can lead in turn to alteration in Aβ secretion through mechanisms that need to be characterized but often involve alternative splicing.

## RMRP

RMRP was first described as the RNA component of the Mitochondrial RNA Processing endoribonuclease, which cleaves the RNA primer for mitochondrial DNA replication (Chang and Clayton 1987). However, this is not an essential function, as complete loss of this activity in yeast did not affect mitochondria (Chang and Clayton 1987). In the nucleolus, RMRP is absolutely required for at least one step in pre-rRNA processing, the cleavage that separates 18S from the 5.8S-28S portion of the pre-rRNA. Human cells lacking MRP RNA accumulate large amounts of pre-rRNA and exhibit a delay in G2 to mitosis progression as well as proliferative arrest (Thiel et al. 2005; Goldfarb and Cech 2017; Vakkilainen et al. 2019). Consistent with such an essential function, it has not been possible to obtain viable *RMRP*-null mice (Rosenbluh et al. 2011). A third described function of RMRP is to constitute the source of at least two short RNAs designated RMRP-S1 and RMRP-S2, produced in a DICER-dependent manner, which themselves regulate a number of genes (Rogler et al. 2014).

RMRP has also been shown to associate with the human telomerase reverse transcriptase catalytic subunit (TERT) to form a complex with RNA-dependent RNA polymerase (RdRP) activity. This activity can form double-stranded RNAs from RNAs with a 3′ fold-back structure. Its main template appears to be RMRP RNA itself, which it extends into a double-stranded RNA that can then be processed into siRNA by DICER (Maida et al. 2009). Indeed, overexpression of RMRP in cells expressing TERT, but not in cells devoid of TERT, led to lower levels of RMRP; this suggests that in TERT-expressing cells, RMRP levels can be lowered by a negative feedback loop involving formation of the RMRP-containing complex with RdRP activity (Maida et al. 2009). Whether this RMRP-containing complex has other functions remains unknown.

### RMRP and cartilage hair hypoplasia

Compound heterozygous or homozygous mutations in the RMRP gene have been found in three types of related diseases, anauxetic dysplasia (AD), cartilage hair hypoplasia (CHH), and metaphyseal dysplasia without hypotrichosis (MDWH), which are collectively referred to as cartilage hair hypoplasia-anauxetic dysplasia spectrum disorders (Ridanpää et al. 2001; Bonafé et al. 2002; Thiel et al. 2005; Makitie and Vakkilainen 2012). All are characterized by short stature due to bone growth deficiency, and for the best understood CHH, sparse hair, and in many cases immunodeficiency, anemia, autoimmunity, allergy, and asthma (Vakkilainen et al. 2018). The observed mutations include insertions or duplications between the TATA box and the TSS, which prevent transcription, and deletions or mutations in highly conserved region of the RNA coding region (Ridanpää et al. 2001; Hermanns et al. 2006). CHH patient cells contain only mutated RMRP. Transcriptional profiling in CHH patient leukocytes showed changes in expression of genes involved in the immune system and cell cycle regulation (Hermanns et al. 2005), whereas a similar analysis in patient fibroblasts defined another set of genes with altered expression including genes involved in PI3K-Akt signaling and cell cycle pathways (Vakkilainen et al. 2019). The two sets contain very few common genes, either because different cell types were examined or as a result of different high-throughput techniques (RNA sequencing versus Affymetrix genome arrays). In both cases, the mechanistic link between RMRP mutations and changes in genes expression is unknown. Altered gene regulation was also observed upon transfection of HEK293 cells with either mimics or inhibitors of RMRP-S1 and RMRP-S2, the two short RNAs derived from RMRP. These two short RNAs, which correspond to RMRP regions affected by mutations causing CHH and whose levels are reduced in CHH patients, regulated very differently sized sets of genes, in both cases containing genes whose function can be related to CHH phenotypes (Rogler et al. 2014).

Targeting a conserved region of *rmrp* in zebrafish and reducing its level led to some CHH phenotypes, as well as to decreased cell proliferation, increased apoptosis, and increased lethality 12–14 d after fertilization. The effect of *rmrp* deficiency was attributed to increased Wnt/β-catenin signaling, as inhibition of Wnt/β-catenin partially alleviated the phenotype of *rmrp* deficit (Sun et al. 2019).

**Figure 5.**
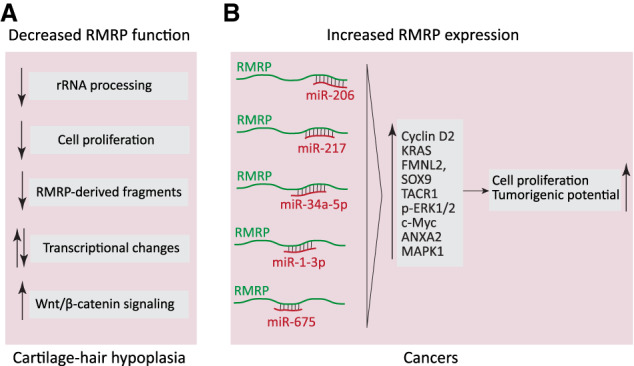
RMRP involvement in CHH and cancer. (*A*) Mutations in the RMRP locus leading to decreased production of functional RMRP cause cartilage hair hypoplasia through various suggested mechanisms including decreased rRNA processing, cell proliferation, and production of RMRP-derived fragments, changes in transcriptome, and increased Wnt/β-catenin signaling. (*B*) Overexpression of RMRP is observed in several cancers, where it is thought to contribute to malignancy by sequestering different miRNAs, and so ultimately increasing the levels of their targets. Such a mechanism was reported for neuroblastoma, hepatocellular carcinoma, cholangiocarcinoma, multiple myeloma, as well as gastric, lung, bladder, and thyroid cancers.

Thus, deficiencies in the Pol III RMRP transcript lead to decreased rRNA processing, decreased production of RMRP-derived fragments, and increased Wnt/β-catenin signaling, both leading to transcriptional changes, as well as decreased cell proliferation, all of which may contribute to the highly variable pleiotropic nature of CHH (Fig. 5).

### RMRP in cancer

Elevated levels of RMRP have been observed in different cancers, in one case as a result of transcriptional activation of the RMRP gene by the Wnt pathway through binding of YAP and β-catenin to the TATA box of the RMRP promoter (Park and Jeong 2015). High RMRP expression has been associated with poor patient prognosis and consistent with this, RMRP knockdown and overexpression experiments in various cancer cell lines revealed a positive role in cell proliferation and tumorigenic potential (Meng et al. 2016; Shao et al. 2016; Cao et al. 2019; Hongfeng et al. 2019; Pan et al. 2019; Tang et al. 2019; Wang et al. 2019a,b; Xiao et al. 2019).

Searches for possible mechanisms by which elevated levels of RMRP might favor tumorigenesis have pointed to RMRP acting as a sponge for certain miRNAs leading to their decreased level, and ultimately to increased levels of their targets (Fig. 5). miR-206 is one such miRNA targeted by RMRP. In gastric cancer cell lines, RMRP overexpression increased Cyclin D2 mRNA levels (Shao et al. 2016), which is a miR-206 target (Zhang et al. 2013). In lung adenocarcinomas and neuroblastomas, RMRP levels were found increased in 71% and 75% of the tumors analyzed, respectively. Overexpression of RMRP in lung adenocarcinoma cells led to decreased levels of miR-206 and, in turn, increased levels of the miR-206 targets KRAS, FMNL2, and SOX9 (Meng et al. 2016). In neuroblastoma and hepatocellular carcinoma cells, decreased miR-206 levels linked to RMRP overexpression led to elevated TACR1, which in turn activated ERK1/2 signaling (Hongfeng et al. 2019; Pan et al. 2019). A negative correlation between RMRP and miR-206 levels was also observed in bladder cancer (Cao et al. 2019).

Other miRNAs besides miR-206 have been identified as RMRP targets. In cholangiocarcinoma cells, knockdown of RMRP led to differential expression of 729 miRNAs as determined by high-throughput sequencing. Among them, miR-217, previously implicated in tumor suppression, was shown to contribute to the oncogenic potential of RMRP (Tang et al. 2019). In multiple myeloma cells, a positive feedback loop consisting of c-Myc, RMRP, and miR-34a-5p was identified. c-MYC was shown to bind to the RMRP promoter and activate its transcription. RMRP RNA acted as a sponge for miR-34a-5p, leading to up-regulation of c-MYC, a target of miR-34a-5p (Xiao et al. 2019). Furthermore, in nonsmall cell lung cancer cells, RMRP overexpression impacted ANXA2 level through binding and repression of miR-1-3p (Wang et al. 2019b), and in papillary thyroid cancer cells, it led to increased MAPK1 levels through binding and repression of miR-675 (Wang et al. 2019a). Unlike decreased RMRP function, increased levels of RMRP thus favor cell proliferation and can enhance expression of various mRNAs through sequestration of various microRNAs.

## BC200

BC200, the shortest long noncoding RNA, was first identified as a neuronal RNA especially abundant in dendrites, which interacted with different proteins and modulated local translation (Sosinska et al. 2015; Samson et al. 2018). Given its neuronal cell localization, it was natural to examine its levels in a neurodegenerative disease such as Alzheimer's disease. A first study reported diminished levels of BC200 in human brains affected by Alzheimer's disease as compared with controls (Lukiw et al. 1992). However, it was later observed that it is global neocortical expression of BC200 that decreased with aging, and that brain areas affected by the disease (Brodmann's area 9) showed increased levels of BC200 RNA (brain cytoplasmic 200 RNA), which correlated positively with its severity (Mus et al. 2007). How increased levels of BC200 might contribute to Alzheimer's disease is unknown.

Although BC200 is most strongly expressed in neural cells, it was also detected in other tissues including testes, ovary, and small intestine (Booy et al. 2017), as well as some, but not all, types of cancers (Chen et al. 1997). For example, it was detected in breast cancer (Iacoangeli et al. 2004) and up-regulated in all nonsmall cell lung cancer tissues (Hu and Lu 2015), in 57% of esophageal squamous cell carcinoma tissues (Zhao et al. 2016), and in many others, some of which are described below. BC200 was found to be required for survival and proliferation of cancerous and noncancerous cells, but to be dispensable for cell cycle arrest and senescence (Booy et al. 2017).

In an effort to understand the role, if any, of BC200 in cancer progression, BC200 levels have been manipulated in cultured cancer cell lines. Although one study reports down-regulation of BC200 in ovarian cancer tissues and in ovarian cancer cell lines (Wu et al. 2016), others have observed elevated levels of BC200 in such tumors and decreased viability of several cancer cell lines, including an ovarian cancer cell line examined by Wu et al. (2016), upon BC200 knockdown (Chen et al. 1997; Booy et al. 2017). Indeed, high levels of BC200 have been generally found to contribute to cell proliferation and/or migration, invasion, and tumorigenic potential. However, little is known about the mechanisms, which appear to vary greatly in different tumors. The paragraphs below summarize observations in various types of cancer and cancer cell lines.

In nonsmall cell lung cancer cells, c-Myc was found to associate with the BC200 promoter and induce its expression, and c-Myc-mediated cell migration and invasion was dependent on BC200. Moreover, BC200 knockdown correlated with lower mRNA and protein levels of the matrix metallopeptidases MMP9 and MMP13, which have been implicated in metastasis (Hu and Lu 2015), as well as with lower levels of PI3K/AKT pathway proteins including PI3K, AKT, and STAT3 (signal transducer and activator of transcription 3) (Gao and Wang 2019). There is, however, no evidence of a direct effect of BC200 on expression of these genes.

In breast cancer, overexpression of BC200 was associated with aggressive tumors. Thus, BC200 was not detected in normal breast tissue or benign breast tumors but was highly expressed in all invasive breast cancer (Iacoangeli et al. 2004) and in peripheral blood of invasive breast cancer patients due to the presence of circulating tumor cells (Iacoangeli et al. 2018). Comparison of BC200 expression in ER-positive versus ER-negative breast cancers further revealed higher expression in the first, which could be linked to binding of ER to an estrogen response element located upstream of the BC200 gene (Singh et al. 2016). However, how does BC200 contribute to cancer progression? Singh et al. (2016) observed that knocking out BC200 by CRISPR/Cas9 reduced cell proliferation in vitro and tumor growth in a xenograft mouse model, and increased apoptosis as well as the levels of the short, proapoptotic form of Bcl-x (Bcl-xS) over the long, antiapoptotic form (Bcl-xL). The mechanism involves association of BC200 RNA with hnRNP A2/B1 and, through a 17-bp complementary sequence, with the 3′ UTR of Bcl-x pre-mRNA, thus impacting alternative splicing and favoring the production of the long form of Bcl-x.

In HeLa cells, knocking down BC200 reduced cell migration and invasion, and significantly altered (over twofold) ribosomal occupancy of 29 mRNAs as determined by ribosome profiling. Many of the more occupied ones encoded histones, whereas many of the less occupied ones encoded cancer-related factors. Among the latter was S100A11, a calcium-binding protein previously implicated in cellular motility of cancer cells. Further experiments confirmed that S100A11 levels were decreased after BC200 knockdown, and the reduced migration capacity of BC200 knockdown cells could be rescued by overexpression of S100A11. This suggests that in HeLa cells, S100A11 is a mediator of the BC200 positive effect on cell migration (Shin et al. 2017).

In colon cancer cells, BC200 knockdown decreased cell proliferation, promoted apoptosis, and reduced the levels of cell cycle factors Cyclin D1, Cyclin E, and c-Myc (Wu et al. 2018). BC200 has been reported to associate with cyclin E2 mRNA and increase its stability, and to associate with cyclin protein E2 and increase E2/CDK2 complex formation (Lin et al. 2018), providing a possible mechanism for this effect. Since Cyclin D1 and c-Myc are targets of the Wnt/β-catenin signal transduction pathway, the protein levels of β-catenin were examined and were indeed found reduced in BC200 knockdown cells. The metalloproteinases MMP-2 and MMP-9, which are involved in cell invasion, were also decreased and the levels of several epithelial-mesenchymal transition markers were altered (Wu et al. 2018).

In oral squamous cell carcinoma, a decrease in esophageal cancer-related gene-4 (*ECRG4*) expression, mediated in large part by methylation of the promoter region, is known to correlate with enlarged tumor size, metastasis, and poor prognosis (Huang et al. 2019, and references therein). Consistent with this, overexpression of *ECRG4* in a tongue squamous cell carcinoma cell line decreased cell proliferation, migration, and levels of the metalloproteinases MMP-9 and MMP-13. In addition, it reduced BC200 levels. The *ECRG4* overexpression-mediated phenotype resulted at least in part from BC200 down-regulation as forced expression of BC200 partially reverted it (Huang et al. 2019).

Other findings include the observation that EpCAM, an oncogene located adjacent to the BC200 gene, is overexpressed together with BC200 in gastric cancers, perhaps reflecting coactivation of these two genes (Ren et al. 2018). Furthermore, BC200 RNA contains seed sequences for miR-138, a miRNA that was reported to inhibit proliferation, migration and invasion in cervical cancer cells (Li et al. 2016; Zhou et al. 2016); indeed, in cervical cancer cells, increased BC200 expression was associated with decreased miR-138 levels (Peng et al. 2018). In colorectal cancer cells, the effects of BC200 knockdown were analyzed by microarrays and identified natriuretic peptide receptor 3 (NPR3) as the gene whose mRNA expression was the most down-regulated. Overexpression of NPR3 in BC200 knockdown cells restored proliferation and inhibited apoptosis, however, whether and how BC200 directly regulated NPR3, and how NPR3 itself impacts on cell proliferation and apoptosis, is completely unexplored (Gu et al. 2018).

## nc886

**Figure 6.**
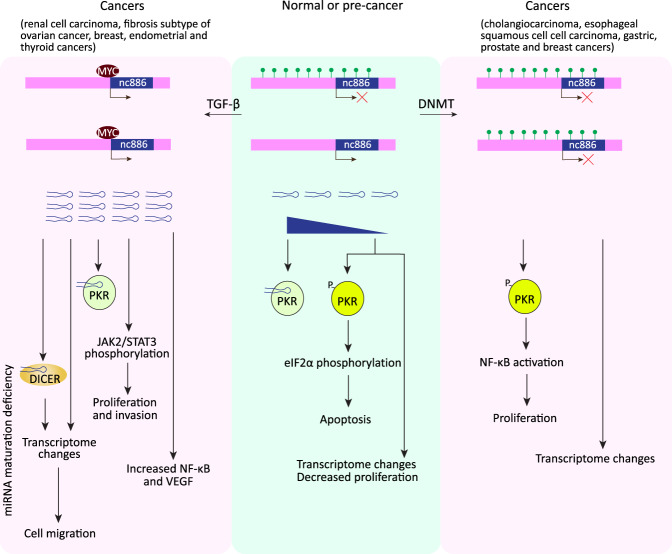
nc886 and cancer. In normal cells, the nc886 locus is methylated in an allele-specific manner but nc886 RNA levels are sufficient to inactivate PKR by direct association. If nc886 levels decrease, PKR can become activated leading to apoptosis, and transcriptional changes can lead to reduced cell proliferation (Kunkeaw et al. 2013; Lee et al. 2014a,b; Lee et al. 2016). In some cancers, nc886 expression is suppressed by progressive DNMT-dependent hypermethylation of its locus, which leads to NF-κB activation and increased cell proliferation as well as to PKR-independent transcriptome changes (Kunkeaw et al. 2013; Lee et al. 2014a,b; Fort et al. 2018). Alternatively, nc886 levels can be increased either by binding of MYC to the demethylated allele (Park et al. 2017) or by TGF-β (transforming growth factor β) activation leading to demethylation of the second allele (Ahn et al. 2018). This has several consequences: increased cell migration as a result of transcriptome changes and as a result of DICER inhibition, also leading to transcriptome changes; inhibition of PKR; increased cell proliferation and invasion through activation of the JAK2/STAT3 pathway; and activation of NF-κB and VEGF (vascular endothelial growth factor) (Hu et al. 2017; Lei et al. 2017; Ahn et al. 2018).

The Pol III nc886 (noncoding RNA 886) gene was previously proposed to encode a pre-miR-886 or an RNA component of the vault complex referred to as vtRNA2-1 (Landgraf et al. 2007; Stadler et al. 2009). However, a later study did not find any evidence that nc886 gives rise to microRNAs nor that it associates with the vault complex (Lee et al. 2011). Instead, this abundant, cytoplasmic RNA was found to associate with, and repress, double-stranded RNA-dependent protein kinase (PKR). PKR is involved in the interferon response to viral infection and is regulated by several proteins and by double-stranded RNAs of viral origin. nc886 is so far the only known cellular RNA that can repress PKR. Activated PKR can phosphorylate eIF2α (eukaryotic translation initiation factor 2) and thus inhibit global translation, leading to apoptosis. However, depending on the cell type and cellular context, it can also activate the NF-κB pathway, which favors cell proliferation (for review, see Lee et al. 2019). In addition, PKR-independent functions of nc886 have been reported (Fig. 6).

Unlike the general trend of Pol III genes to be overexpressed in cancer cells, nc886 transcription tends to be decreased in many types of cancer including cholangiocarcinoma (eight out of 16 tumors analyzed) (Kunkeaw et al. 2013), esophageal squamous cell carcinoma (54 out of 84 tumors analyzed with a decrease of at least twofold) (Lee et al. 2014a), and gastric and prostate (six out of six tumors analyzed) cancers (Lee et al. 2014b; Fort et al. 2018). The nc886 gene is located within a CpG island and in all cases examined, down-regulation resulted from progressive methylation of the gene flanking sequences. In contrast, nc886 expression was increased in renal cell carcinoma (Lei et al. 2017) and in thyroid (11 out of 37 tumors analyzed with an overexpression of >1.3, which tend to be the more aggressive tumors) (Lee et al. 2016), endometrial (Hu et al. 2017), and some ovarian (Ahn et al. 2018) cancers. In human mammary epithelial cell lines that serve as models of early breast tumorigenesis, decreased or increased levels of nc886 were observed depending on the cell line, in the first case due to CpG hypermethylation and heterochromatin induction, and in the second case due to enhanced binding of MYC near the nc886 locus (Park et al. 2017).

In nonmalignant cholangiocytes and in nc886-expressing cholangiocarcinoma cells, nc886 knockdown led to up-regulation of PKR activity with two different outcomes: In nonmalignant cells, activated PKR resulted in increased eIF2α phosphorylation, decreased protein synthesis, and promoted apoptosis, whereas in cholangiocarcinoma cells, it resulted in activation of NF-κB (Kunkeaw et al. 2013). A similar differential effect was observed upon acute knockdown of nc886 RNA in human gastric nonmalignant (HFE-145) or malignant (SNU-601 and SNU-683) cell lines (Lee et al. 2014b). In addition however, upon nc886 knockdown in nonmalignant HFE-145 cells or in both nonmalignant and malignant esophageal cells, gene expression arrays detected increased expression of several oncogenes (including FOS, NF-κB targets, MYC) as well as of genes involved in the interferon, innate immune response, and inflammation pathways (Lee et al. 2014a,b). Reduced nc886 expression as a result of methylation was also observed in various prostate cancer samples, but in this case, reduced expression correlated with increased expression of some prostate cancer cell cycle progression genes (Fort et al. 2018).

In cancers with increased levels of nc886, several effects were reported upon nc886 knockdown. In an endometrial cancer cell line, it led to activation of PKR, increased levels of caspase-3, increased apoptosis, decreased levels of NF-κB, VEGF, and decreased cell proliferation (Hu et al. 2017). In a renal cell carcinoma cell line, nc886 knockdown similarly led to increased apoptosis and decreased cell proliferation, whereas overexpression led to increased proliferation and invasion apparently in part through activation of the JAK2/STAT3 pathway, as treatment of the cells with a JAK2 inhibitor attenuated the nc886 overexpression-derived phenotypes (Lei et al. 2017).

In some ovarian cancer cell lines, nc886 was reported to be silenced by hypermethylation, which also affected the nc886 neighboring gene *TGFBI*, a known TGF-β induced gene. TGF-β treatment led to hypomethylation of the nc886 locus and induction of its expression together with that *TGFBI*. TGF-β treatment enhanced cell migration, and this effect was dependent on nc886 induction. In fact, ectopic expression of nc886 also enhanced cell migration, and 273 genes were commonly altered by TGF-β treatment and nc886 ectopic expression. Furthermore, nc886 was shown to interact with DICER and inhibit maturation of many miRNAs, suggesting that nc886 is the factor constituting the previously observed link between TGF-β activation and miRNA suppression in a subtype of ovarian cancer (“integrated mesenchymal” or the “fibrosis” subtype) (Ahn et al. 2018).

PKR-independent effects of nc886 have also been observed in another study. Successive deletions of the PKR gene and then nc886 by CRISPR/Cas9 in an immortalized but not fully transformed thyroid cell line (Nthy-ori) resulted in decreased proliferation, migration, and invasion compared with both the corresponding WT and PKR KO cells, and in specific deregulation of 201 genes compared with the PKR KO cells, many of which are involved in tumorigenesis and apoptosis (Lee et al. 2016).

Together, these results indicate that nc886 expression levels can greatly affect cell proliferation and survival, and illustrate how the effects vary depending on cell type and cellular context. The nc886 RNA can play both PKR-dependent and PKR-independent roles (Fig. 6), and PKR itself is regulated by several other factors. Thus, the precise function of nc886 in different cellular environments and the complete mechanisms involved remain to be determined.

## Conclusion

In this review, we have deliberately focused on links between human diseases and defects in Pol III transcription. Diseases linked to Pol III transcription defects can so far be separated into two categories. In the first are those clearly caused by decreased and/or partially defective Pol III transcription due to mutations in components of the Pol III transcription apparatus (Pol III-related leukodystrophies, growth failure, and central nervous system anomalies, abnormally severe varicella-zoster infections), or to defective synthesis of a specific Pol III product (neurodegeneration due to mutation in the tRNA ^Arg^_UCU_ gene, cartilage hair hypoplasia). In the second category are those (various types of cancer) linked to deregulation of several Pol III products, for which deregulation contributes to the disease but is not necessarily the only, or the original, cause. In both cases, we need more research to understand the full breadth of changes in Pol III transcripts associated with the disease, and by which mechanisms such changes affect the organism.

We need to know more about how different Pol III genes are differentially regulated, in particular how silent Pol III genes are activated under specific circumstances. For example, some tRNA genes, which are silent in the mouse liver, become activated during liver regeneration (Yeganeh et al. 2019). SINEs, which have long been considered as junk DNA, are specifically expressed in response to heat shock and repress Pol II transcription (Allen et al. 2004; Mariner et al. 2008). Additionally, as we see above, BC200, normally silenced in most tissues, is activated in different cancers (Chen et al. 1997; Shin et al. 2018). However, in each case, the exact mechanisms leading to activation still elude us. Mostly, we need better methods to quantify precisely specific Pol III transcripts, including methods able to distinguish different tRNA isoacceptors, and methods able to quantify not only primary Pol III products but also mature, fully modified and functional Pol III products (Orioli 2017). Knowing the full extent of Pol III transcriptome deregulation in the various disorders will go a long way toward providing a more complete understanding of how Pol III deregulation contributes to diseases.
